# Living with ankylosing spondylitis: an open response survey exploring physical activity experiences

**DOI:** 10.1093/rap/rkz016

**Published:** 2019-06-27

**Authors:** Peter C Rouse, Martyn Standage, Raj Sengupta

**Affiliations:** 1 Department for Health, Centre for Motivation and Health Behaviour Change, University of Bath, Bath, UK; 2 Rhuematology, Royal National Hospital for Rheumatic Diseases, Royal United Hospitals Bath, Bath, UK

**Keywords:** ankylosing spondylitis, physical activity, motivation, barriers, facilitators

## Abstract

**Objective:**

The aim was to gather in-depth, rich accounts of physical activity experiences of people living with AS, to include symptom management, consequences for symptoms, factors that encourage and disrupt physical activity, and motivations that underpin participation in physical activity.

**Methods:**

Participants (*n* = 149; 60% female) completed a Bristol Online Survey that consisted of open questions to capture rich qualitative data. In total, 96% of participants self-reported having AS (1% other arthritis; 3% missing), and 51% had this diagnosis for >20 years. A content analysis was conducted to identify the key themes/factors from within the open question responses. A frequency analysis was used to ascertain the most commonly identified themes and factors.

**Results:**

Fifty different physical activities were participated in over the previous month. Physical activity can improve and worsen arthritis symptoms, and fluctuations in participation exist even in the most active. Pain and fatigue were the two most frequently identified factors that stopped people with AS from being physically active. Participants reported more autonomously driven motivations than controlled motivations for participating in physical activity.

**Conclusion:**

People with AS can and do participate in a diverse range of physical activities, but fluctuations in activity levels occur owing to disease- and non-disease-specific factors. Individually tailored plans and self-monitoring are important to optimize levels of physical activity and maximize benefits for people living with AS. Multiple reasons why AS patients participate in physical activity were revealed that included both adaptive (i.e. autonomous) and maladaptive (i.e. controlled) forms of motivation.

**Key messages**

- People living with AS can and do live physically active lifestyles.
- Individually tailored plans and self-monitoring can optimize levels of physical activity and maximize benefits in people living with AS.
- People living with AS indicated multiple autonomous and controlled motivations for participating in physical activity.

## Introduction

AS is a chronic inflammatory arthritis that causes pain, fatigue and compromised physical functioning, leading to low health-related quality of life [1, 2]. The typically early adulthood onset of AS highlights the long-term physical, psychological and financial cost to both patients and society. Physical activity has been identified as a core strategy for the treatment of AS symptoms [3–5] and has been shown to provide benefits above and beyond that of pharmaceutical intervention (e.g. improved mobility) [4, 6]. Physical activity may also be a particularly effective treatment option compared with other forms of arthritis owing to its influence on the axial system [3].

Research has shown physical activity to improve mobility, pain, physical function and disease activity in people with AS [4, 7]. A consensus statement has recently been published to provide evidence-based guidance for practitioners working with AS patients who wish to accrue the treatment benefits [3]. Despite these recent advancements in the literature, the majority of people with AS fail to participate in sufficient quantities of physical activity and/or to maintain participation for long enough to accrue the evidenced benefits [8, 9]. Indeed, long-term adherence to physical activity remains one of the greatest challenges in this population. Therefore, understanding the experiences of people who live with AS is a crucial component in redressing this challenge [3].

People with AS share many of the barriers and facilitators to physical activity experienced by the general population, yet also face additional disease-specific factors that influence physical activity behaviour [10]. It is also conceivable that the challenges people with AS experience differ from those of other, more frequently studied arthritis conditions, such as OA and RA, partly as a result of the disease targeting the spine and the subsequent restriction of mobility. Pain, fatigue, stiffness and disability all represent prominent disease-specific challenges to people who live with AS [9, 11, 12]. In a study that revealed the barriers and facilitators to participating in physical activity, O’Dwyer *et al.* [13] conducted semi-structured interviews until no new knowledge was being obtained. Their findings revealed that 17 middle-aged AS participants, recruited in Dublin, with a range of disease severity (BASFI range 0–6.2) viewed physical activity as a means to improve their AS symptoms and general health but, importantly, helped to assist in other aspects of daily living, such as driving and sporting performance. Factors were also identified that hindered participation in physical activity, including negative attitudes, lack of knowledge in relevant others, symptoms of AS and lack of resources. A study investigating the experience of Danish men living with AS revealed a fear of becoming dependent on others to handle everyday life and not being able to play with children [14]. However, physical activity acted as a positive release from living with a chronic disease and provided a ‘small sense of happiness’ or a ‘mental boost’ even though a fear of putting too much strain on the body remained [14]. These qualitative accounts provide rich data about the attitude towards physical activity of Danish and Irish people living with AS, but the generalizability is limited owing to the small number of studies and cases. No research we are aware of has identified the experiences of patients living with AS in the UK or considered the motivations of people to participate in physical activity.

Theoretical frameworks of motivation have been used to understand how to support the adoption and maintenance of physical activity in a variety of populations, including people with rheumatic conditions [15, 16]. For example, self-determination theory [17] proposes that the quality of motivation (i.e. the reason why) carries the greatest consequences for the adoption and maintenance of behaviours, such as physical activity, in addition to the psychological wellbeing benefits that can be achieved. Specifically, autonomous motivations (i.e. a personal interest or enjoyment of an activity or personally valuing the benefits associated with a behaviour) have been shown to support participation in physical activity and enhanced psychological wellbeing [16, 18]. In contrast, controlled motivations that are driven by external forces and contingencies support burn-out and negative psychological outcomes [19, 20]. Despite the apparent relevance of self-determination theory to the adoption and maintenance of physical activity for people living with AS, little research has investigated the propositions of the theory in this context.

The aim of this study was to gather accounts of physical activity experiences of people living with AS, including symptom management, consequences for symptoms, factors that encourage or disrupt physical activity, and the motivations that drive participation. In order to capture data from a larger number of people who live with AS, an online survey composed of open questions was used. This methodology might help people with AS to provide a full and less biased account owing to reduced feelings of social desirability [21] because of the anonymity provided by completing the survey online. These data will provide an invaluable source to guide clinical practice and a future research agenda that is driven by the true needs and experiences of those people living with AS.

## Methods

A purposive sample was collected via the National Ankylosing Spondylitis Society (United Kingdom) distributing a link to the online survey to their members (*n* = 3731; [22]). Only when participants had read an information sheet and ticked to indicate informed consent could they complete the survey. A total of 149 participants completed the survey over a 6-week period (October–November 2017). Participants were on average 48 (S.D. 14) years of age, and 60% were female. In total, 96% of participants self-reported having AS (1% other arthritis; 3% missing), and 51% had had this diagnosis for >20 years. No measure of disease activity was taken.

A Bristol Online Survey was developed containing questions that explored the experiences of people living with AS and in line with the existing literature [13] (see Table 1 for example questions). The survey was developed and reviewed by an expert panel comprising a consultant rheumatologist and two academics from a Center of Motivation and Health Behaviour Change. Ethical approval was obtained from the University ethics board REACH (reference no. EP 17/18 124).

**Table 1:** Open-ended questions within online survey

What do you do to manage your arthritis?
What types of activities do you currently participate in (i.e. in the past month)?
What things stop you or make it difficult for you to participate in regular physical activity?
Is there anything that encourages you to be active?
How does participating in physical activity worsen or improve your arthritis?
Why do you participate in physical activity (i.e. what reasons)?

**Fig. 1:**
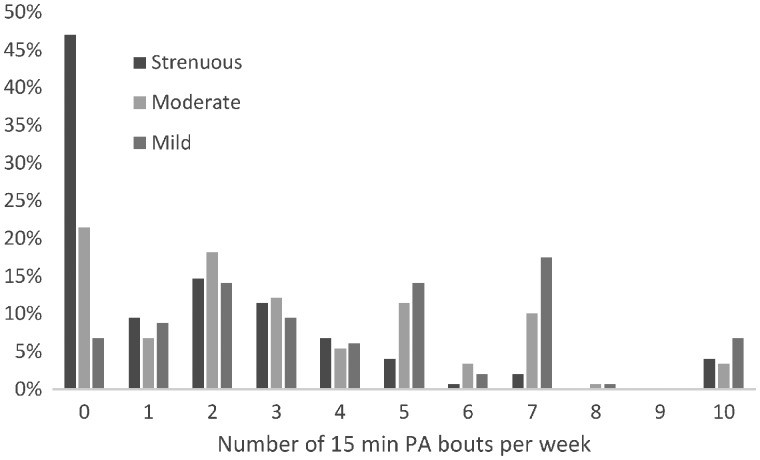
Frequency (percentage) of 15 min bouts of strenuous, moderate and mild physical activity

The Godin Leisure Time Physical Activity Questionnaire [23] was included within the survey, which is an assessment of physical activity that has been used in past research with participants having other rheumatic diseases (e.g. RA) [16]. This self-report measure asks participants to identify the frequency of 15 min bouts of strenuous-, moderate- and mild-intensity activity over the past 7 days. A total weekly leisure time physical activity index is calculated using the following formula: (9 × strenuous) + (5 × moderate) + (3 × mild) [16]. Fig. 1 indicates the percentage of participants who reported different numbers of physical activity bouts (15 min) per week for three different intensities (i.e. 9% of our sample reported participating in one bout of strenuous PA per week). In total, 53% of participants participated in at least one bout (15 min) of strenuous physical activity, 79% in at least one bout of moderate activity and 93% in at least one bout of mild activity.

### Analyses

A qualitative manifest content analysis was conducted on the participants’ responses to the open-ended questions [24]. Initially, all survey responses were read individually by the primary author to provide a sense for the data. Once familiarization with the data was achieved, responses were coded inductively. Once coding had finished, the primary and second authors re-read and compared the codes with the original text. Consensus about the codes was established via a panel of experts who included expert qualitative researchers [25] and a consultant rheumatologist. This method of analysis allows realistic conclusions to be drawn and stays close to the words used by participants. A frequency analysis was subsequently conducted to identify the most commonly identified themes.

## Results

### Management of AS symptoms

Medication (77%) and staying physically active (85%) were the two most identified techniques adopted to manage AS symptoms. However, 25 other techniques were also reported, including use of heat and ice (12%), having a healthy diet (8%), hydrotherapy (7%), rest/sleep (6%) and pacing yourself (3%). When asked who they looked to for help and support in the management of symptoms, participants revealed a wide social network. In this regard, rheumatologists and general practitioners were most frequently used for support, but other targets included partners, friends, AS charities, physiotherapists, AS patients, pharmacists and online forums. In contrast, when asked about where they looked for information about their arthritis, the internet (52%) and the National Ankylosing Spondylitis Society website (48%) were dominant compared with the rheumatology team (18%), general practitioner (3%) or physiotherapist (2%). Other sources of information identified included support groups, other arthritis-based charities and the National Health Service website.

### Advice about physical activity

In total, 68% of the sample stated that they knew the physical activity guidelines for their arthritis, and 82% indicated that they had been provided with advice about participating in physical activity. Yet, the majority had never been referred to an exercise or physical activity programme (55%).

### Types of activities

Despite walking (62%), swimming (22%) and cycling (17%) being the most prominent, a total of 50 different activities were identified that had been participated in over the past month, including dancing, horse riding, climbing, pickleball, curling and trampolining (see Table 2).

**Table 2:** The frequency of the most commonly cited activities participated in during the last month

Activity	Frequency (%)
Walking	62
Swimming	22
Cycling	17
Pilates	15
Exercise class	13
Running	11
Gym	10
Yoga	9
Weights	7
Gardening	7
Hydrotherapy	7
Spin	6
Physio	5
Manual labour	5
Stretching	5
Dancing	3
Hiking	3
Aqua class	3
Housework	3
Cross-training	3
Body weights	2
Tennis	2
Rowing machine	2
Football	2
Play	2
Bowling	1

### Worsen or improve arthritis

**Fig. 2:**
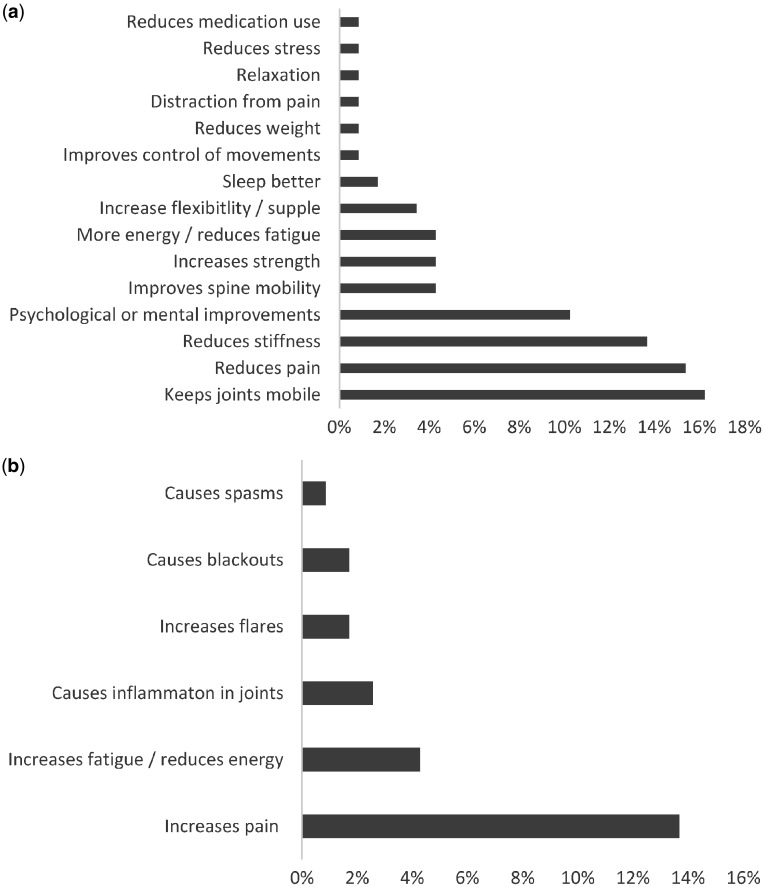
Participant-identified ways that physical activity improves and worsens AS symptoms (**a**) Ways that physical activity improves AS symptoms and the percentage of participants who identified each factor. (**b**) Ways that physical activity worsens AS symptoms and the percentage of participants who identified each factor.

When asked about the effect that physical activity has on their arthritis, slightly >79% stated that it either slightly or definitely improved their AS symptoms. In contrast, 17% stated that physical activity slightly or definitely worsened their symptoms. Participants self-reported 21 different ways that physical activity improved or worsened their arthritis, with 15 of those related to ways that physical activity improved their arthritis (see Fig. 2a and b). Respondents highlighted that physical activity keeps their joints mobile (16%), reduces stiffness (14%) and enhances psychological and mental wellbeing (10%).

*‘I feel better when I exercise, my hips are less stiff and I sleep better at night.’*

*‘Improves my stamina, smoothness of movement, eases any pain.’*

Pain relief was identified as a way that physical activity improved symptoms (15%), but at the same time 14% of our participants suggested that physical activity can increase pain.

Although reported less, other ways in which physical activity made symptoms worse included increased fatigue or reductions in energy (3%), causing inflammation in joints (2%), causing blackouts (1%) and increases in flares (1%).

*‘It worsens me because I never know when to stop, resulting in grossly over doing the activity.’*

The over-riding theme from this question, however, was that physical activity can both worsen and improve their arthritis. In total, 34% of respondents highlighted that an optimal level of physical activity was needed to ensure that physical activity had a positive rather than negative impact on their arthritis.

*‘I feel as though I can move more freely after the exercise and it does improve, however, on occasions I suffer terribly the next day for days afterwards and have not energy to get out of bed in the morning.’*

### What makes participation difficult?

**Fig. 3:**
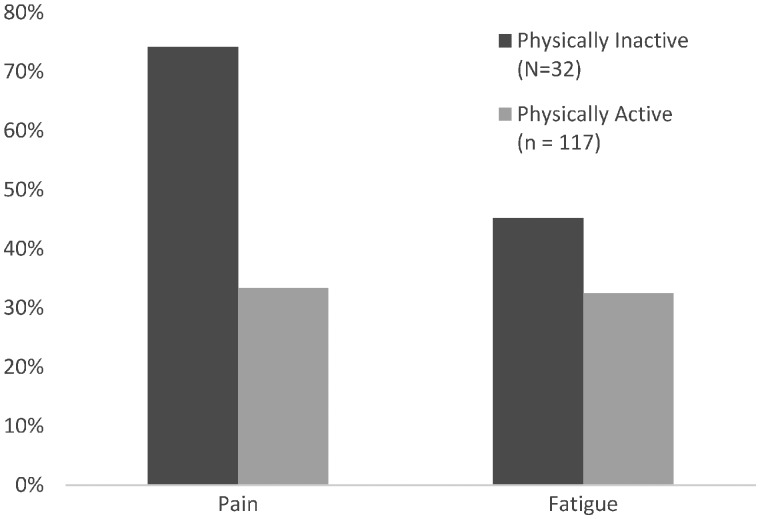
Frequency (percentage) of physically active *vs* not physically active individuals identifying pain and fatigue as barriers

The following responses are separated into those subjects who identified themselves as physically active (*n* = 117) and those who did not (*n* = 32). Despite identifying themselves as physically active, pain (32%) and fatigue (33%) were the two most frequently identified factors that stopped people with AS from being as active as they would like (see Fig. 3). However, 22 other factors were also identified, including stiffness (9%), lack of mobility (9%), flares (3%) and illness (2%). A lack of time (9%), work (8%), cost (3%) and childcare (3%) were the most prominent non-disease-related barriers.

Similar to their physically active counterparts, participants who self-identified as not being physically active (*n* = 32) indicated that pain (74%) and fatigue (45%) were the most prominent factors preventing participation in physical activity out of the 14 factors identified. Additional disease-specific factors included fused joints (10%), limited movement (16%) and stiffness (6%). Fear of harm, lack of guidance and low self-esteem were each identified by one person. Non-disease-related factors included time (13%), work (10%) and family (6%) commitments.

### What encourages physical activity?

A wide range of factors (35 in total) that encouraged participation were identified by those who were physically active on a regular basis. These included knowing the benefits of participating in regular physical activity (12%), having the support of friends and family (11%), belonging to a support group (9%), reductions in pain (9%) and enjoying the activity (6%). A total of 86% of physically active respondents indicated that their level of physical activity fluctuated from one day to another. Of the 21 different causes of fluctuations identified, fatigue (30%), pain (18%) and work (9%) were the three most frequently cited factors. Other causes of fluctuations in physical activity were the weather, stiffness, family commitments and sedentary behaviour.

The non-physically active participants (*n* = 32) identified 12 factors that encouraged them to participate in physical activity, including support from a knowledgeable person (13%), wanting to spend time with children (10%) and knowledge of benefits (10%). However, these non-physically active participants most frequently reported that ‘nothing’ encouraged them to be physically active (19%).

### What are the different motives for AS patients to be physically active?

Twenty-six different reasons for participating in physical activity were reported. Consistent with the dynamic nature of motivation for physical activity [28], the majority of participants identified several reasons for being physically active. The predominant reason was to manage and control symptoms of AS (43%), followed by gaining general health benefits (18%), keeping fit (12%) and enjoyment of the activity (12%). The reasons identified by the participants were categorized into the five different motivational regulations as proposed by self-determination theory [17] and are shown in Table 3. These categorizations showed participants to have and endorse eight more autonomously driven forms of motivation than controlled types. For example, our sample indicated that they personally valued the ability to control AS symptoms through regular physical activity,

*‘To keep muscles and tendons stretched and better able to move ankylosed joints’,*

that physical activity contributed to their identity,

*‘I have been trampolining for 12 years—it is part of my identity’*

and because they enjoy the activity.

*‘Mainly because I enjoy being physically active even if I did not suffer with arthritis’.*

In contrast, some reported more controlled and externally driven reasons, such as fear of disability,

*‘I have already seen some places where things were bad enough that I had to work hard to return to a better quality of life. I do not wish to return there so I get out and I move’,*

because someone else has told them that it will help with their disease management,

‘Told it would help my condition’

or out of feelings of guilt.

*‘Guilt because my first Rheumatologist (before NSAIDs) said there was little that could be done or given to me but I must keep moving as much as I could whatever the pain, and here I am now at 80, still trying to do as he said’.*

**Table 3:** Reasons identified for participating in physical activity categorized as autonomous or controlled forms of motivation

Reason identified	Frequency (%)	Motivation	
Social life	5	Intrinsic	Autonomous motivations
Time alone	1	Intrinsic	
Identity	2	Integrated	
Joint movement	20	Identified	
Health benefits	18	Identified	
Control symptoms/manage AS	17	Identified	
Wellbeing/mental	17	Identified	
Keep fit	12	Identified	
Reduce pain	5	Identified	
Strength	4	Identified	
Muscle flexibility	3	Identified	
Increase energy	3	Identified	
Healthy for family	2	Identified	
Reduce stress	1	Identified	
Relaxation	1	Identified	
Sleep better	1	Identified	
Concentration	1	Identified	
Fear of disability	7	Introjected	Controlled motivations
Guilt	1	Introjected	
Live longer	1	Introjected	
Keeps me going	1	Introjected	
Weight management	5	Extrinsic	
Work/study	4	Extrinsic	
Dog	1	Extrinsic	
Competition	1	Extrinsic	
Keep busy/distraction	1	Extrinsic	

## Discussion

Despite evidence indicating that the majority of people with AS are not sufficiently active to gain health benefits [8, 9], our findings revealed that people with AS self-reported that they can and do regularly participate in a wide range of physical activities. Nevertheless, people with AS who are physically active typically experience fluctuations in levels of participation owing to a range of factors, including fatigue and pain. Similar factors that disrupt physical activity behaviour were identified by their non-physically active counterparts, but they appear to lack the desire or resources to overcome these barriers. Participants also clearly identified that optimal levels of participation in physical activity are needed to ensure that beneficial treatment effects are accrued and that disease symptoms are not aggravated.

Along with pharmaceutical methods, physical activity was one of the most important methods of disease management. The wide range of methods identified, including heat and ice, healthy diet and rest, emphasizes the importance of multi-component treatment programmes. The rheumatology team were most frequently relied upon for help and support in managing the symptoms of AS; however, the internet was where our participants most frequently looked for information about their arthritis. These findings confirm previous research highlighting that the internet might be a viable and important medium to offer further help and support [14].

Our sample of AS patients had a high level of knowledge about physical activity, with 68% indicating that they know the recommended guidelines and 82% reporting that they had received advice about being physically active. This knowledge is supported by the high levels of physical activity that our sample self-reported. Yet, despite this knowledge, the majority had not been referred to an exercise or physical activity programme, indicating a reliance on the individual to organize their own activity.

The majority of participants indicated that physical activity can improve AS symptoms. The improvements identified focused on better mobility and function and on enhanced mental or psychological wellbeing. An important addition made by this work in terms of informing the extant literature, and for people living with AS, is the number and diversity of activities that are being conducted on a regular basis. Example activities reported included dancing, aqua classes, cross-training, tennis, trampolining and bowling. This diversity highlights the ability of people diagnosed with AS to continue activities previously enjoyed and can offer encouragement to others about continued quality of life after an AS diagnosis. Experiences of people with AS who regularly participate in activities could help to empower others who would like to participate in physically demanding activities but are fearful or unsure if they will be able to do so.

Similar to other forms of arthritis, disease-related factors, such as pain and fatigue, act as both barriers and facilitators that lead to fluctuations in the quantity of physical activity being conducted [26]. Of particular note is that for physically active respondents the experience of pain acts as a barrier to physical activity but reductions in pain encourage participation. In contrast, the non-physically active participants only identified pain as a barrier. The identification that physical activity can both improve and worsen arthritis symptoms, such as pain, indicates the importance of individually tailored programmes. AS patients need to work collaboratively with their health-care team and social support networks to understand their body and AS symptoms to optimize the quantity and intensity of physical activity both during flares and when symptoms are low. Self-monitoring and body awareness appear to be crucial when understanding the optimal amount to improve arthritis symptoms and not exacerbate them. Although these findings confirm that programmes should be individually tailored, they raise questions about the propositions of previous qualitative research about whether participation in physical activity should be maximized when symptoms are low [13]. Research is needed to explore whether maximizing or increasing the quantity or intensity of activity on ‘good days’ is the best approach. The reported fluctuations in physical activity also indicate that the typical cross-sectional and even longitudinal research designs might fail to capture these variations. Therefore, future person-centred study designs and analyses might offer greater insight into the antecedents and consequences of physical activity within AS.

Motivation is often a neglected but theoretically and empirically supported factor [17, 27] that accounts for the reasons why people engage in physical activity behaviour. This study provides a unique insight into the reasons that drive physical activity behaviour in AS patients by using open-ended questions rather than a structured questionnaire. Results revealed a wide range of reasons that drove participation in physical activity and included both autonomous and controlled forms of motivation. In this sample, the number of autonomously motivated reasons for participating in physical activity outnumbered the controlled reasons. However, it was interesting to note that the majority of participants had multiple and varied motivations for participating in physical activity. Such a finding is consistent with the tenets within self-determination theory, and the dynamic interplay among multiple forms of motivation and their relationship with disease-related and wellbeing outcomes is worthy of future research attention [28].

It is also important to distinguish this research from traditional methods of qualitative research, such as interviews and focus groups that allow greater exploration of the points raised and may provide a richer and more personal account of each persons’ experience. However, this research drew on the strengths of an online survey in order to reach a larger number of people whilst using open questions to gather rich, non-prescriptive answers. Despite this aim, it is important to acknowledge that physical activity levels in our sample were higher than in other studies [16] and could be attributed to the purposive recruitment strategy via NASS. This indicates that our sample might not be typical of people diagnosed with AS and that they already endorsed autonomous forms of motivation to use physical activity to manage disease symptoms, thus making it difficult to generalize these findings. This assumption is supported by the bias towards female respondents (60%). AS typically affects males three times as often as females [29]. The benefit of this non-representative sample is the revelation that people with AS can participate in regular physical activity and in very different and diverse ways.

People with AS can live physically active lifestyles, as evidenced by the diverse range of activities reported in this study. Yet, even those who participate in physical activity on a regular basis experience fluctuations in activity levels owing to a range of disease- and non-disease-related factors. Individually tailored plans developed in close collaboration with a wide support network including health professionals and close self-monitoring are important to optimize levels of physical activity and maximize physical activity benefits for people living with AS.

*Funding*: No specific funding was received from any funding bodies in the public, commercial or not-for-profit sectors to carry out the work described in this manuscript.

*Disclosure statement*: The authors have declared no conflicts of interest.
